# Precision oncology: AI’s breakthrough in TNBC chemoresistance prediction

**DOI:** 10.1097/MS9.0000000000003790

**Published:** 2025-08-28

**Authors:** Anjani Mahesh Kumar Cherukuri, Maliha Khalid, Muhammad Talha, Aminath Waafira

**Affiliations:** a Department of Surgery, Guntur Medical College, Guntur, India; b Jinnah Sindh Medical University, Karachi, Pakistan; c King Edward Medical University, Lahore, Pakistan; d The Maldives National University, Malé, Maldives

**Keywords:** artificial intelligence, histopathology, neoadjuvant chemotherapy, triple-negative breast cancer

## Abstract

Triple-negative breast cancer (TNBC) is an aggressive subtype with limited targeted therapies and variable response to neoadjuvant chemotherapy (NAC), with only 30–40% of patients achieving optimal outcomes. Predicting NAC resistance at diagnosis is a critical unmet clinical need to avoid unnecessary toxicity and delays in alternative treatments. Artificial intelligence (AI) applied to digitized histopathology offers a promising solution. Recent advances include NACNet, a spatial graph convolutional network incorporating tumor microenvironment context with high predictive accuracy; morphometric analysis using convolutional neural networks to identify histological signatures of resistance; and ensemble deep learning frameworks guided by expert cognition to enhance generalizability across multi-center datasets. While current models face challenges in external validation and require broader datasets to minimize bias, these developments demonstrate AI’s growing role in individualized treatment planning for TNBC. By integrating biological insight with advanced computational modeling, AI holds potential to revolutionize chemoresistance prediction and precision oncology.

*To the Editor,*

Triple-negative breast cancer is an aggressive subtype of breast cancer. There are limited targeted therapies for TNBC, and TNBC often displays a heterogeneous response to neoadjuvant chemotherapy (NAC). Only 30–40% of TNBC patients respond well to NAC, while the remaining patients either respond moderately or are refractory to NAC^[1]^. Although NAC provides significant clinical benefits, such as tumor downstaging before surgery, it is often associated with a substantial decrease in the patient’s quality of life, attributed to its adverse effects^[2]^. Owing to this, for non-responders, NAC introduces unnecessary toxicity, delays potentially more effective treatments, and may lead to overtreatment. This poses a critical and unmet need, and accurately predicting NAC response at diagnosis remains a major clinical challenge.

Artificial intelligence (AI), particularly deep learning applied to digitized histopathology, is emerging as a promising avenue to meet this challenge. Li et al. introduced NACNet, a histology context-aware transformer graph convolutional network that captures spatial interactions within the tumor microenvironment to predict NAC response. By modeling the complex tissue architecture rather than isolated image tiles, NACNet achieved a high accuracy of 90% and an area under the curve (AUC) of 0.82. This approach illustrates how integrating biological context can markedly enhance predictive power in identifying NAC-resistant TNBC^[3]^.

Krishnamurthy et al. focused on extracting morphometric patterns from hematoxylin and eosin-stained biopsies using an unsupervised deep convolutional neural network. Their model achieved an overall AUC of 0.75 in predicting pathological complete response, with higher accuracy in early-stage disease. This indicates AI’s ability to detect subtle histological signatures associated with resistance^[4]^.

A major drawback for AI models is generalizability beyond their training data. Addressing this, Yang et al. developed an ensemble deep learning framework guided by expert cognition to enhance external validation performance. Their model improved AUC from approximately 61.5% to 67.8% on multi-center external datasets, moving closer to AI tools that can be reliably applied across diverse clinical settings^[5]^.

These studies demonstrate AI’s potential in predicting NAC resistance in TNBC by combining biological insight with advanced modeling and validation techniques. While NACNet’s spatial graph approach leads in accuracy, Yang et al.’s ensemble deep learning framework offers a promising path for broader applicability. Krishnamurthy et al.’s morphometric analysis further supports AI’s role in early and accurate response prediction^[4,5]^.

Despite the promising results, current AI models generally achieve less accurate AUCs, indicating the need for further refinement before routine clinical use. Expanding diverse datasets and standardizing validation protocols will be essential to minimize bias and enhance the robustness of the results. Despite the limitations, AI’s capacity to integrate complex tissue features offers a powerful tool to address the unmet clinical need of predicting chemotherapy resistance in TNBC, paving the way for individualized treatment decisions. Our work is in line with the TITAN Guidelines on the need for transparency in AI use in healthcare^[6]^.

## Data Availability

No data were generated for this manuscript.

## Peer and provenance statement

This article was not commissioned and was externally peer-reviewed.
